# Insulin resistance and levels of adipokines in patients with untreated early rheumatoid arthritis

**DOI:** 10.1007/s10067-015-3106-8

**Published:** 2015-11-03

**Authors:** Sara Manrique-Arija, Inmaculada Ureña, Pedro Valdivielso, José Rioja, Francisco G. Jiménez-Núñez, María V. Irigoyen, Antonio Fernández-Nebro

**Affiliations:** UGC de Reumatología, Instituto de Investigación Biomédica de Málaga (IBIMA), Hospital Regional Universitario de Málaga, Universidad de Málaga, Málaga, Spain; UGC de Medicina Interna, Instituto de Investigación Biomédica de Málaga (IBIMA), Hospital Universitario Virgen de la Victoria, Universidad de Málaga, Málaga, Spain; Departamento de Medicina y Dermatología, Instituto de Investigación Biomédica de Málaga (IBIMA), Universidad de Málaga, Málaga, Spain

**Keywords:** Adipokines, DMARDs, Early rheumatoid arthritis, Insulin resistance

## Abstract

The aim of this study is to investigate the presence of insulin resistance (IR) in patients with untreated early rheumatoid arthritis (ERA) and its relationship with adipokines, inflammatory cytokines, and treatment. In this prospective study, we enrolled 46 ERA patients with a disease duration of <1 year, and 45 sex-, age-, race-, and body mass index (BMI)-matched controls. Patients and controls with diabetes or a history of glucocorticoid (GC) or disease-modifying antirheumatic drugs (DMARDs) use were excluded. Patients were assessed at the time of diagnosis (visit 1) and after 6 months of treatment (visit 2). The main outcomes were homeostatic model assessment of IR (HOMA-IR) and β-cell function (HOMA-β) and quantitative insulin sensitivity check index (QUICKI). A multivariate regression analysis was performed to analyze IR adjusting according to lipids, body composition, physical activity, nutrition, and inflammatory cytokine and adipokine levels. The baseline HOMA-IR, HOMA-β, and QUICKI values were similar in both groups. However, patients showed lower levels of physical activity, total cholesterol, and high-density lipoprotein. Moreover, the inflammatory cytokines and resistin concentrations were higher in patients than controls. Multivariate analysis indicated that BMI and baseline rheumatoid factor levels were positively associated with HOMA-IR and HOMA-β, and negatively with QUICKI. After DMARD treatment, patients showed improvements in inflammatory parameters and lipids whereas IR remained stable. Furthermore, adiponectin and resistin concentrations decreased slightly. Our data suggest that IR is not present in ERA patients either at diagnosis or at 6 months after treatment. However, symptom duration and fat mass appear to be related.

## Introduction

Rheumatoid arthritis (RA) is a chronic inflammatory disease associated with premature mortality and multimorbidity [1–4]. The cardiovascular risk of RA patients is comparable to that of patients with diabetes [1]. Both traditional and nontraditional risk factors contribute to accelerated atherogenesis [5, 6], including systemic inflammation, GC use, dyslipidemia, hypertension, age, diabetes, and insulin resistance (IR) [5, 7, 8]. The development of systemic inflammation in RA results in energy metabolism disorders, microvascular dysfunction, disability, and physical inactivity [9].

Adipokines, such as leptin, resistin, and adiponectin, critically link nutritional status, physical activity, metabolism, inflammation, and immunity. Similarly, adipokines modulate, and are modulated, by inflammatory cytokines. However, the complex relationships between atherogenesis, cytokines, and adipokines are not yet fully understood [1].

IR is not a disease unto itself, but rather a physiological abnormality that greatly increases the chances of developing diabetes and metabolic syndrome [2]. Although RA patients have a high prevalence of metabolic syndrome and factors related to obesity, dyslipidemia, and metabolism disorders, only few studies have been specifically designed to investigate IR in this population. In addition, most of the studies have been conducted in RA patients with longstanding illness, a severe inflammatory burden, comorbidities, and high intake levels of medications that influence the metabolism, including IR [3–7]. Moreover, the relationships among insulin secretion, lipid profiles, proinflammatory cytokines, and adipokines have not been studied in depth in untreated early RA (ERA) patients.

Although certain evidence states that IR is increased in long-term RA cases, it is not clear whether the development of IR accompanies the onset of symptoms or instead occurs subsequently due to poor disease control in some patients. In the present study, we aimed to determine whether patients with ERA have increased IR, and to analyze the relationships among factors known to affect glucose metabolism at ERA diagnosis (visit 1) and after 6 months of treatment with DMARDs (visit 2).

## Materials and methods

### Study design and setting

This is a prospective longitudinal 6-month observation study, which was conducted in the University Hospital of Malaga, Spain. Patients were assessed when ERA diagnosis was made and before beginning treatment with DMARDs or GC (visit 1) and after 6 months of treatment (visit 2). The study was approved by the medical ethics committee, and all subjects provided written informed consent before enrollment.

### Subjects

#### Patients

We enrolled consecutive, untreated ERA patients who were referred to our rheumatology outpatient clinic. The inclusion criteria were as follows: a symptom duration at diagnosis of <12 months, age of ≥16 years, presence of active disease (DAS28 ≥ 3.2), and diagnosis of RA according to the American College of Rheumatology/European League Against Rheumatism 2010 classification criteria [8]. The exclusion criteria were the presence of any inflammatory or rheumatic disease other than RA (except for secondary Sjögren’s syndrome), diabetes or impaired glucose tolerance (IGT), or any noncontrolled general condition. Subjects with active infections, those who were pregnant or breastfeeding, and those with current or previous use of oral antidiabetic drugs, insulin, GC, or DMARDs were excluded. However, patients who were receiving nonsteroidal anti-inflammatory drugs (NSAIDs) were not excluded.

Subjects who had been taking beta-blockers, angiotensin-converting enzyme inhibitors, angiotensin receptor blockers, or lipid-lowering drugs for at least 3 months were included if the dose was the same for at least 28 days.

#### Controls

Control subjects, were pair-matched 1:1, by sex, age, race, and body mass index (BMI), were selected among nonrheumatic patients from associated primary care centers, who volunteered for the study. The control subjects were those who did not meet the classification criteria for any inflammatory disease. The inclusion/exclusion criteria for control subjects were identical to those used for the patients.

### Outcome measures

The outcome measures were IR measured by the homeostatic model assessment of IR (HOMA-IR), the homeostatic model assessment of β-cell function (HOMA-β) [9], and the quantitative insulina sensitivity check index (QUICKI) [10].

### Other recorded variables and study protocol

Subjects were assessed through a standardized clinical interview in visit 1 and visit 2.

Weight, height, BMI (weight/height2), blood pressure (the mean of two measurements obtained 5 min apart after subjects had rested in a supine position for 10 min), waist and hip circumferences, and waist-to-hip ratio (WHR) were all measured [10]. The data recorded included the traditional Framingham risk factors, current and previous treatment details, physical activity level, and body composition as measured by dual energy X-ray absorptiometry (DEXA; GE Lunar Prodigy enCORETM 2006 software).

Also, fat mass index (FMI) and free-fat mass index (FFMI) were measured.

Patients and controls completed self-administered semi-quantitative questionnaires about their educational level, diet, and physical activity at work and leisure. To detect IGT, subjects with baseline blood glucose levels of <126 mg/dL underwent an oral glucose tolerance test (OGTT) in visit 1. Patients and controls with IGT in OGTT were excluded. Samples were collected after 12–16 h of fasting between 9:00 and 10:00 AM.

Enzymatic methods were used to measure the levels of total cholesterol (TC), triglycerides, and high-density lipoprotein (HDL) cholesterol. In addition, low-density lipoprotein (LDL) cholesterol concentrations were calculated [11]. The serum insulin levels at baseline and 2 h after loading were measured by an enzyme-amplified chemiluminescent assay (Immulite ONE^®^). The serum levels of high-sensitivity C-reactive protein (hsCRP) were measured by standard nephelometry (normal range, 0–6 mg/L). The serum levels of tumor necrosis factor alpha (TNF-α) were measured using an automated immunoassay analyzer (Immulite®, Diagnostic Products Corporation, Los Angeles, CA; normal range, 0–8.1 pg/mL). The serum interleukin (IL)-6 levels were measured by an enzyme chemiluminescent assay (QuantiGlo^®^; normal range, 0–5.84 pg/mL). Adiponectin (normal value, mean ± standard deviation [SD], 11.5 ± 5.9 μg/mL), resistin (normal value, median [interquartile range {IQR}], 7.2 [5.4–8.5] ng/mL), and leptin levels were measured with ELISA kits (Mediagnost^®^; normal values were considered to fall between the 5th and 95th percentiles, after adjusting for sex and BMI, according to the manufacturer’s recommendations).

Variables of RA included symptom duration, swollen joint count (in 28 joints), tender joint count (in 28 joints), and patient’s assessment of disease activity (measured on a 0–100 mm visual analog scale). The disease activity score in 28 joints (DAS28) [12] was calculated, and all patients completed a health assessment questionnaire (HAQ) [13]. The patients were managed according to the Clinical Practice Guideline for the Management of RA in Spain (GUIPCAR 2007) [14] and followed up by a rheumatologist in routine clinical practice.

### Definitions

IR was defined as a HOMA-IR score of ≥2.75 based on the 90th percentile of healthy people in a Spanish cohort [15]. IGT and diabetes were diagnosed in accordance with the recommendations of the American Diabetes Association [16]. Smoking was only considered in active smokers. Hypertension was defined as a blood pressure of ≥140/90 mm Hg, or ongoing treatment with antihypertensive medications [17].

Obesity was defined according to BMI [18]. Dyslipidemia (LDL cholesterol >160 mg/dl, triglycerides >200 mg/dl, and HDL cholesterol <40 mg/dl (men) and <50 mg/dl (women)) and metabolic syndrome were defined according to the NCEP ATP III [19, 20]. The estimated cardiovascular risk of fatal cardiovascular disease within 10 years was calculated using the SCORE model. A cutoff point of SCORE ≥5 % was used [21]. The presence of inflammatory activity was defined as a DAS28 score of ≥3.2 [12]. FMI was defined as fat mass (kg)/height squared (m^2^) and FFMI as fat-free mass (kg)/height squared (m^2^). The value of fat mass and fat-free mass was obtained by DEXA [22].

### Statistical analyses

The sample size was calculated assuming a prevalence of insulin resistance of 19 % in controls and 51 % in patients with recent onset RA [23] (RR = 2.7) and an α and β error of 0.05 and 0.20, respectively. The estimated initial sample was 44 subjects per group, but the sample size increased further assuming that approximately 10 % of patients may be finally excluded from analysis for not meeting inclusion criteria.

Data are presented as means (SD), medians (IQR), or totals with percentages. The baseline characteristics were compared between groups using the *χ*^2^ test, the Student’s two-tailed *t* test (Fisher’s exact test when necessary), or the Mann–Whitney *U* test. Paired comparisons of changes from baseline to 6 months in RA patients were performed with the paired *t* test or Wilcoxon matched-pairs signed rank test, as appropriate. Changes in the categorical variables were evaluated by McNemar’s test.

Bivariate correlations were calculated between quantitative variables. The main significant variables were selected as independent variables for multiple linear regression models (backward stepwise elimination), wherein the HOMA-IR, HOMA-β, or QUICKI was used as dependent variable. As HOMA-IR, HOMA-β, or QUICKI are not normally distributed, a Box-Cox transformation was performed before introduce them in the multivariate analysis. The analysis was performed using the Methodological and Statistical Assessment Unit of FIMABIS (Malaga, Spain) using The R Statistical software (version 3.1.1). The other analyses were performed with SPSS 21.0 software (IBM Corp, USA). Two-tailed tests and a 5 % significance level were used in all analyses.

## Results

### Baseline characteristics

The study population comprised 56 Caucasian patients with early arthritis. During follow-up, seven patients were excluded because they had arthritis other than RA, and another three patients were excluded because of previously unknown diabetes mellitus.

None of the subjects had IGT. The final analyzed data set included 46 ERA patients and 45 control subjects.

Table 1 lists the baseline characteristics of ERA patients and controls. Although there were no significant differences between ERA patients and controls in the majority of the baseline variables, ERA patients were more likely to have a smoking history (*p* = 0.035) and lower levels of TC and HDL cholesterol than the control subjects (*p* = 0.024 and *p* = 0.044, respectively). Despite elevated systemic inflammation, people with RA were not significantly more insulin resistant than controls [HOMA-IR, median (IQR) = 1.0 (0.3–2.6) in ERA patients vs 0.9 (0.4–1.8); *p* = 0.515]. The prevalence of IR was 21.7 % (95 % CI, 12.3 to 35.6) in ERA patients and 15.6 % (95 % CI, 7.7 to 28.8) in control subjects, the difference between which was not significant (*p* = 0.592). There were also no major differences in any carbohydrate metabolism parameters between patients and controls, except for a slightly higher 2-h OGTT result in ERA patients than in control subjects (*p* = 0.036) but the values are within the normal range in both groups. The concentrations of TNF-α, IL-6, and resistin were significantly higher in ERA patients than in the control subjects (*p* < 0.001 for TNF-α and IL-6, and *p* = 0.008 for resistin), but there were no differences in adiponectin and leptin concentrations between these groups.

**Table 1:** Baseline characteristics in patients and controls (visit 1)

	ERA patients	Controls	*p* value
Age (years), mean (SD)	51.6 (17.6)	50.7 (12.7)	0.787
Gender: female, *n* (%)	35 (76.1)	33 (73.3)	0.763
Time until diagnosis ERA in months, mean (SD)	5.9 (3.5)	–	–
Smoking, *n* (%)	12 (26.1)	4 (8.9)	0.035
Baseline hypertension, *n* (%)^a^	15 (32.6)	7 (15.6)	0.057
Anthropometric features			
BMI (kg/m^2^), mean (SD)	26.8 (4.6)	27.1 (3.5)	0.673
Waist circumference (cm), mean (SD)	89.7 (14.6)	90.0 (11.4)	0.909
Hip circumference (cm), mean (SD)	104.5 (13.1)	106.0 (6.6)	0.484
Waist-hip ratio, mean (SD)	0.85 (0.09)	0.84 (0.09)	0.572
Fat mass index, mean (SD)	10.49 (4.25)	10.52 (3.25)	0.830
Fat-free mass index, median (IQR)	15.48 (13.88–16.89)	15.03 (13.86–17.30)	0.848
Metabolic features			
Total cholesterol (mg/dl), mean (SD)	195.5 (40.3)	214.8 (39.5)	0.024
Triglycerides (mg/dl), mean (SD)	110.3 (60.9)	106.7 (45.8)	0.750
LDL cholesterol (mg/dl), mean (SD)	122.8 (33.4)	137.0 (34.8)	0.053
HDL cholesterol (mg/dl), mean (SD)	52.2 (14.8)	59.1 (16.7)	0.044
TG/HDL ratio, mean (SD)	1.8 (1.2–3.0)	2.0 (1.0–2.9)	0.585
TC/HDL ratio, mean (SD)	3.97 (1.09)	3.91 (1.34)	0.819
Dyslipidemia, *n* (%)	36 (78.3)	39 (86.7)	0.292
Fasting glucose (mg/dl), mean (SD)	84.5 (9.9)	80.9 (13.8)	0.165
2 h OGTT glucose (mg/dl), mean (SD)	113.9 (35.3)	99.1 (30.9)	0.036
Metabolic syndrome, *n* (%) NCEP ATP III	7 (15.2)	5 (11.1)	0.563
Insulin (μIU/ml), median (IQR)	4.5 (1.5–11.9)	4.2 (1.6–8.5)	0.821
HOMA-IR, median (IQR)	1.0 (0.3–2.6)	0.9 (0.4–1.8)	0.515
HOMA-IR ≥2.75, *n* (%)	10 (21.7)	7 (15.6)	0.592
HOMA-β-cell, median (IQR)	16.8 (4.3–45.9)	14.3 (5.1–35.2)	0.943
QUICKI, mean (SD)	0.39 (0.07)	0.40 (0.07)	0.549
QUICKI ≤0.33, *n* (%)	10 (21.7)	8 (17.8)	0.793
Acute phase reactants, cytokines and adipokines			
hsCRP (mg/l), median (IQR)	12.3 (7.1–23.2)	1.6 (1.1–2.1)	<0.001
ESR (mm/h), mean (SD)	32.9 (21.4)	7.2 (5.4)	<0.001
TNF-alpha (pg/ml), median (IQR)	10.7 (8.7–12.8)	6.9 (6.1–7.9)	<0.001
IL-6 (pg/ml), median (IQR)	20.1 (5.2–46.0)	1.2 (0.7–1.9)	<0.001
Adiponectin (μg/ml), mean (SD)	10.4 (6.4)	9.2 (5.6)	0.352
Resistin (ng/ml), median (IQR)	7.0 (5.7–10.0)	5.4 (4.6–7.9)	0.008
Leptin (ng/ml), mean (SD)	17.2 (17.7)	16.1 (10.9)	0.744

*ERA* early rheumatoid arthritis, *ND* No data, *BMI* body mass index, *LDL* low-density lipoprotein, *HDL* high-density lipoprotein, *TG/HDL* triglycerides/high-density lipoprotein, *TC/HDL* total cholesterol/high-density lipoprotein, *OGTT* oral glucose tolerance test, *NCEP ATP III* National Cholesterol Education Program Adult Treatment Panel III, *HOMA IR* insulin resistance index, *HOMA β-cell* β-cell function index, *QUICKI* Quantitative Insulin Sensitivity Check Index, *hsCRP* high-sensitivity C reactive protein, *ESR* erythrocyte sedimentation rate, *TNF-alpha* tumor necrosis factor alpha, *IL-6* interleukin-6

^a^BP ≥140/90

#### Educational level, diet, drugs, and physical activity

The control subjects had higher educational level than ERA patients (58.5 vs 24.4 % received a university education, *p* = 0.014). With regard to diet, although ERA patients ate fish more frequently than control subjects (51.2 vs 15.6 % ate fish at least three times weekly, *p* = 0.004), no difference was detected for other foods (i.e., eggs, dairy, fruits, vegetables, meats, sausages, sweets, snacks, or alcohol).

Notably, ERA patients showed a lower baseline recreational physical activity level than control subjects (subjects who had never done exercise: 40 vs 11.6 % [*p* = 0.002]; subjects who only occasionally had done exercise: 33.3 % vs. 65.1 % [*p* = 0.002]).

At baseline, 20 (43.5 %) ERA patients were taking NSAIDs on demand, of which 15 (75 %) were being treated with ibuprofen, 3 (15 %) with naproxen, and 2 (10 %) with diclofenac; 1 (2.2 %) control subject was taking NSAIDs (*p* < 0.001), whereas the remaining patients took acetaminophen with or without an opioid. No differences in the use of other drugs were noted between groups.

Only five patients (10.9 %) and seven controls (15.6 %) were taking lipid-lowering drugs, and no significant difference was observed between them (*p* = 0.509). After a reanalysis excluding patients taking lipid-lowering drugs, we did not find changes in the results.

Educational level, diet, and physical activity did not show differences.

### Baseline relationship between RA risk factors and insulin sensitivity in ERA patients

Table 2 shows the manner in which key RA baseline variables correlated with HOMA-IR, HOMA-β, and QUICKI. A positive correlation was noted between the HOMA-IR score and age, duration of RA symptoms, rheumatoid factor (RF) levels, antibodies to cyclic citrullinated peptide (anti-CCP) levels, TNF-α levels, CT/HDL ratio, and parameters related to adiposity. HOMA-β score showed essentially the same associations as the HOMA-IR score. QUICKI showed an inverse association with the same variables that correlated with the HOMA-IR score.

**Table 2:** Bivariate correlations of patient characteristics with HOMA-IR, HOMA-B, and QUICKI

VARIABLE	HOMA-IR Spearman *ρ*	HOMA-B Spearman *ρ*	QUICKI Spearman *ρ*/*r*
Age (years)	0.422**	0.431*	−0.417
Duration of symptoms (months)	0.370*	0.368*	−0.358*
Rheumatoid factor (IU)	0.380*	0.390*	−0.380*
Anti-CCP antibodies (IU)	0.308*	0.252*	−0.308*
DAS28	0.014	0.056	−0.085
hsCRP (mg/L)	0.055	0.068	−0.055
ESR (mm/h)	0.035	0.049	−0.095
BMI (kg/m^2^)	0.449**	0.456**	−0.443**
Waist circumference (cm)	0.483**	0.476**	−0.450**
Hip circumference (cm)	0.432**	0.429**	−0.329*
Waist-hip ratio	0.376*	0.349*	−0.322*
Total fat (kg)	0.555***	0.564***	−0.487**
Total free-fat (kg)	0.205	0.183	−0.029
Total mass (kg)	0.430*	0.400**	−0.353
Systolic blood pressure (mm Hg)	0.141	0.149	−0.256
Total cholesterol (mg/dl)	0.177	0.206	−0.198
LDL cholesterol (mg/dl)	0.198	0.211	−0.239
HDL cholesterol (mg/dl)	−0.193	−0.221	0.263
Triglycerides (mg/dl)	0.152	0.232	−0.272
TG/HDL ratio	0.243	0.317*	−0.243
CT/HDL ratio	0.317	0.356*	−0.380*
Adiponectin (μg/ml)	−0.095	−0.044	−0.010
Resistin (ng/ml)	0.139	0.197	−0.139
Leptin (ng/ml)	0.253	0.279	−0.286
TNF-alpha (pg/ml)	0.292*	0.284*	−0.292*
IL-6 (pg/ml)	− 0.002	0.050	−0.093

*HOMA IR* insulin resistance index, *HOMA β-cell* β-cell function index, *QUICKI*, Quantitative Insulin Sensitivity Check Index, *Anti-CCP* anti-cyclic citrullinated peptide, *DAS28* Disease Activity Score in 28 Joints, *hsCRP* high-sensitivity C reactive protein, *ESR* erythrocyte sedimentation rate, *BMI* body mass index, *LDL* low-density lipoprotein, *HDL* high-density lipoprotein, *TG/HDL* triglycerides/high density lipoprotein, *TC/HDL* total cholesterol/high-density lipoprotein, *TNF-alpha* tumor necrosis factor alpha, *IL-6* interleukin-6

*p < 0.05; **p > 0.005; ***p < 0.001

The resistin level was positively associated with the IL-6 level (*ρ* = 0.376; *p* = 0.022), but negatively associated with TC (*ρ* = −0.362; *p* = 0.013) and HDL cholesterol (*ρ* = 0.380; *p* = 0.012) levels. Furthermore, the leptin level showed a positive correlation with weight, BMI (*r* = 0.717; *p* < 0.001), total mass (*r* = 0.480; *p* = 0.001), total fat (*r* = 0.795; *p* < 0.001), and waist (*r* = 0.463; *p* = 0.001) and hip (*r* = 0.636; *p* < 0.001) circumferences. Total adiponectin level shows no correlation with any of the above variables.

Furthermore, the TNF-α, but not IL-6 level, was positively correlated with BMI (*ρ* = 0.364; *p* = 0.013), waist (*ρ* = 0.342; *p* = 0.020) circumference, WHR (*ρ* = 0.392; *p* = 0.007), triglycerides/HDL ratio (*ρ* = 0.388; *p* = 0.010), TC/HDL ratio (*ρ* = 0.415; *p* = 0.006), and triglyceride levels (*ρ* = 0.335; *p* = 0.023), fasting glucose levels (*ρ* = 0.313; *p* = 0.034), and fasting insulin levels (*ρ* = 0.291; *p* = 0.049). No correlation between TNF-α level and DAS28 score was noted.

Table 3 shows the multivariate linear regression models for HOMA-IR, HOMA-β, and QUICKI. According to these analysis models, both total fat and symptom duration were the only predictors of the HOMA-IR, HOMA-β, and QUICKI scores.

**Table 3:** Multivariate linear regression models for HOMA-IR (Box-Cox), HOMA-B (Box-Cox), and QUICKI (Box-Cox) in ERA patients

Predictor	B	95 % CI for B	*p* value	*R* ^2^
Dependent variable: HOMA-IR^a, b^				0.409
Total fat mass (kg)	0.00005	0.00002 to 0.00009	0.001	
Symptom duration (months)	0.105123	0.009682 to 0.200564	0.032	
Dependent variable: HOMA-B^b, c^				0.371
Total fat mass (kg)	0.000047	0.000016 to 0.000077	0.004	
Duration of symptoms (months)	0.096256	0.003768 to 0.188745	0.042	
Dependent variable: QUICKI^c^				0.413
Total fat mass (kg)	−0.000008	−0.000013 to −0.000004	0.001	
Duration of symptoms (months)	−0.015484	−0.029685 to −0.001284	0.034	

^a^Independent variables: age, total fat, waist circumference, duration of symptoms (from onset of symptoms to RA-diagnosis), TNFα, RF, and TC/HDL ratio

^b^Box-Cox transformed: Lambda values used for HOMA-IR, HOMA-B, and QUICKI, were −0.151, −0.104, and 0.125, respectively

^c^Independent variables: age, total fat, waist circumference, duration of symptoms (from onset of symptoms to RA-diagnosis), TNFα, RF, TC/HDL ratio, and TG/HDL ratio

### Six-month follow-up examination in ERA patients

Table 4 shows the characteristics of ERA patients at baseline and after 6 months.

**Table 4:** Characteristics of the ERA patients at baseline (visit 1) and after 6 months (visit 2)

Variable	Baseline	6 months	*p* value
Arthritis characteristics			
Disease duration (months), mean (SD)	5.9 (3.5)	–	
No. of swollen joints (28 assessed)	8 (4–15)	1 (0–3)	<0.001
No. of tender joints (28 assessed)	4 (2–11)	0 (0–2)	<0.001
Patient’s assessment of disease activity (VAS, mm), mean (SD)	66.3 (20.9)	33.9 (24.5)	<0.001
hsCRP (mg/l), median (IQR)	12.3 (7.1–23.2)	5.6 (3.0–9.9)	<0.001
ESR (mm/h), mean (SD)	32.9 (21.4)	18.7 (12.0)	<0.001
RF (IU/l), median (IQR)	43.8 (17.9–90.5)	20.2 (10.0–80.5)	0.071
Anti-CCP (IU), median (IQR)	108 (3.8–341)	171.0 (1.6–340.0)	0.884
DAS28, mean (SD)	5.5 (1.3)	3.2 (1.4)	<0.001
DAS28 <3.2, *n* (%)	0 (0)	32 (69.6)	<0.001
DAS28 <2.6, *n* (%)	0 (0)	16 (34.8)	<0.001
HAQ, mean (SD)	1.3 (0.7)	0.548 (0.567)	<0.001
Treatment			
NSAIDs, *n* (%)	20 (43.5)	40 (86.9)	<0.001
Glucocorticoids, *n* (%)	–	4 (9.6)	
Doses (mg/day), median (IQR)		5.0 (3.1–10.6)	
Dose maximum (mg/day), mean (SD)		14.4 (11.3)	
Methotrexate, *n* (%)	–	43 (93.5)	
Doses (mg/week), median (IQR)		17.5 (15.0–20.0)	
Methotrexate plus Leflunomide, *n* (%)		1 (2.2)	
Methotrexate plus sulfasalazine, *n* (%)		1 (2.2)	
Sulfasalazine, *n* (%)	–	1 (2.2)	
Anthropometric features			
BMI, mean (SD)	26.8 (4.7)	27.1 (5.3)	0.061
Fat mass index, mean (SD)	10.49 (4.25)	10.63 (4.36)	0.107
Fat-free mass index, median (IQR)	15.48 (13.88–16.89)	14.85 (13.58–16.30)	0.411
Metabolic features			
Total cholesterol (mg/dl), mean (SD)	195.5 (40.3)	195.0 (38.9)	0.977
Triglycerides (mg/dl), mean (SD)	110.3 (60.9)	116.1 (63.8)	0.441
LDL cholesterol (mg/dl), mean (SD)	122.8 (33.4)	118.6 (31.2)	0.300
LDL cholesterol >100 mg/dl, *n* (%)	31 (67.4)	15 (32.6)	0.006
HDL cholesterol (mg/dl), mean (SD)	52.2 (14.8)	56.2 (14.6)	0.010
Low HDL cholesterol, *n* (%)	13 (28.3)	9 (19.6)	0.289
TG/HDL ratio, mean (SD)	1.9 (1.2–3.0)	1.6 (1.4–2.0)	0.289
TC/HDL ratio, mean (SD)	3.83 (1.05)	3.52 (0.72)	0.084
Fasting Glucose (mg/dl), mean (SD)	84.5 (9.9)	83.3 (10.7)	0.412
Fasting Insulin (μIU/ml), median (IQR)	4.5 (1.5–11.9)	5.0 (1.5–10.2)	0.876
HOMA-IR, median (IQR)	1.0 (0.3–2.6)	1.0 (0.3–1.9)	0.981
HOMA-IR ≥2.75, *n* (%)	10 (21,7)	8 (17.4)	0.754
HOMA-β-cell, median (IQR)	16.8 (4.3–45.9)	18.5 (3.4–47.6)	0.791
QUICKI, mean (SD)	0.39 (0.07)	0.40 (0.07)	0.477
QUICKI ≤0.33, *n* (%)	10 (21.7)	8 (17.4)	1.000
Cytokines and adipokines			
TNF-alpha (pg/ml), median (IQR)	10.6 (8.7–12.8)	7.8 (6.7–9.5)	<0.001
High TNF-alpha, *n* (%)	37 (80.4)	15 (32.6)	0.004
IL-6 (pg/ml), median (IQR)	20.1 (5.2–46.0)	2.9 (1.4–8.6)	<0.001
High IL-6, *n* (%)	32 (70.3)	15 (32.6)	<0.001
Adiponectin (μg/ml), mean (SD)	10.4 (6.4)	9.0 (5.0)	0.001
Resistin (ng/ml), median (IQR)	7.0 (5.7–10.0)	5.6 (4.6–8.8)	0.002
Leptin (ng/ml), mean (SD)	17.2 (17.7)	21.28 (23.2)	0.091
Cardiovascular risk			
SCORE (≥5 %)	45 (97.8)	45 (97.8)	1.000

*ERA* early rheumatoid arthritis, *VAS* visual analogic scale, *hsCRP* high-sensitivity C reactive protein, *ESR* erythrocyte sedimentation rate, *RF* rheumatoid factor, *Anti-CCP* anti-cyclic citrullinated pPeptide, *DAS28* Disease Activity Score in 28 Joints, *HAQ* Health Assessment Questionnaire, *NSAIDs* nonsteroidal anti-inflammatory drugs, *LDL* low-density lipoprotein, *HDL* high-density lipoprotein, *TG/HDL* triglycerides/high-density lipoprotein, *TC/HDL* total cholesterol/high-density lipoprotein, *HOMA IR* insulin resistance index, *HOMA β-cell* β-cell function index, *QUICKI* Quantitative Insulin Sensitivity Check Index, *TNF-alpha* Tumor necrosis factor alpha, *IL-6* interleukin-6, *SCORE* Systemic Coronary Risk Evaluation model

Approximately 76 % of the cases were RF-positive and 72 % were anti-CCP-positive.

No patients had extra-articular disease manifestations or a history of GC or DMARD use. All patients exhibited disease activity. One patient was lost to follow-up. After the baseline evaluation, ERA patients received treatment according to their rheumatologist’s criteria (Table 4). All ERA patients received escalated dosages of methotrexate, and 36 (56.5 %) of these patients received a tapering regimen of concomitant GC (median [IQR] initial dose, 10 [5–20] mg/day). Patients treated with GC had a higher DAS28 score at baseline (mean [SD] 6.0 [1.3] vs 4.7 [0.9]; *p* = 0.001) and worse HAQ score (1.519 [0.822] vs 0.984 [0.456]). We did not find any correlation between the GC and the level of insulin resistance measured by HOMA –RI (Spearman’s rho 0.123; *p* = 0.406). During the 6-month follow-up, one patient switched to sulfasalazine, and two patients received combined treatment. At 6 months, 40 patients (86.9 %) were treated with NSAIDs, 25 (62.5 %) with ibuprofen, 9 (22.5 %) with naproxen, 4 (10 %) with indomethacin, and 2 (5 %) with diclofenac. Most patients (32; 80 %) were taking NSAIDs on demand. After 6 months of treatment, approximately 70 % of the ERA patients had a low DAS28 score. In addition, there was a slight improvement of physical activity (patients who do exercise: 32.6 % at baseline vs 56.5 % after 6 months; McNemar’s test, *p* = 0.091), which was closer to the levels of physical activity noted in the controls (65.1 %). There were no significant differences between the anthropometric variables, blood pressure, SCORE risk and metabolic characteristics, HOMA-IR, HOMA-β, QUICKI, fasting glucose, and fasting insulin levels at baseline and at 6-month evaluation (Table 4). However, after 6 months of treatment, we observed an improvement in lipid profiles, whereas the median concentrations of cytokines and adipokines changed. Adiponectin, resistin, TNF-α, and especially IL-6 levels showed a marked decline. In contrast, leptin concentrations were slightly, but not significantly, increased.

None of the parameters mentioned above were affected by GC treatment after 6 months, with the exception of larger reductions in IL-6 (mean [SD] −38.5 [45.8] vs −11.3 [19.2] pg/mL; *p* = 0.028), hsCRP (−21.2 [31.8] vs −1.6 [9.1] mg/L; *p* = 0.008), and ESR (−17.4 [20.2] vs −6.3 [11.7] mm/h; *p* = 0.049), as compared to ERA patients who did not take GC.

## Discussion

We noted that there were no differences in the HOMA-IR or HOMA-β scores, or QUICKI between ERA patients and matched control subjects.

Several studies have shown that RA patients have impaired fasting insulin sensitivity [3, 7, 23–25]. However, most of these studies were either conducted in patients with longstanding, treated RA or involved non-BMI-matched controls. Therefore, it is not clear whether this impaired insulin sensitivity was present from the time of RA development or if it developed over time in association with lifestyle changes, persistent inflammation, drug exposure, and other factors.

To our knowledge, few studies have investigated the presence of IR in ERA [25–27].

One of these was a noncontrolled study that included 196 patients treated with DMARDs and/or GC [26]; therefore, their results cannot be compared with our findings. The other study included 66 untreated RA patients with a mean disease duration of 9.8 months and 40 sex- and age-matched controls [25]; this study indicated that HOMA-IR score was higher in ERA patients than in the control subjects. However, these results may differ from the findings of the current study because of several important methodological reasons, such as the presence of fewer control subjects than cases, a longer disease duration (9.8 vs 5.9 months in our study), a significantly higher BMI in ERA patients than in control subjects, and the lack of investigation of impaired glucose tolerance in the previous study; in addition, fat mass and body fat distribution were not evaluated. A sedentary lifestyle, overweight/obesity, central adiposity, and impaired glucose tolerance are widely known to be associated with IR [28]. Furthermore, Sahin et al. did not study the effect of both the evolution time and early treatment of ERA on the IR.

The third one is a cross-sectional study [27]. We also found some differences from our study, the most important being a longer disease duration (9.2 vs 5.9 months in our study). Moreover, they did not study impaired glucose tolerance, other adipokines and cytokines, body composition, diet, or exercise. Furthermore, the effect of the evolution time and early treatment of ERA on the Insulin resistance was not analyzed. The only predictors of IR identified in the present study were total fat and RA symptom duration. These results suggest that IR may not be present during the few first months of arthritis but may develop subsequently if poorly controlled inflammatory disease persists and if there is an increase in the amount of fat mass.

This increase in fat mass might be explained in part by the presence of lower physical activity, which is associated with pain and physical dysfunction caused by arthritis. In fact, the ERA patients in the present study reported a low baseline level of recreational physical activity, which improved when arthritis was controlled. These results are consistent with those found in previous studies [29, 30].

There is considerable evidence that supports the association between IR and the presence of increased proinflammatory cytokine levels in longstanding RA [3, 4, 24, 25]. Nevertheless, the only proinflammatory factor that showed some correlation with IR in the present study was TNF-α. This cytokine impairs insulin sensitivity and is linked to obesity and diabetes [24]; moreover, it downregulates tyrosine kinase activity and interferes with signaling pathways [31]. According to our results, TNF-α might exert an even greater and faster influence on metabolic processes than IL-6 because, in addition to its previously reported correlation with IR, it is also correlated with central adiposity and lipid profiles [31].

Besides proinflammatory cytokines, adipokines produced by fat tissue may also have effects on glucose homeostasis, appetite, and inflammatory responses [32, 33].

Consistent with results of previous studies, our results did not show differences in leptin and adiponectin concentrations between ERA patients and control subjects [34, 35]. In fact, leptin concentrations in the ERA patients in the present study were positively correlated with anthropometric variables and IR, but not with inflammatory parameters. In contrast, the resistin concentration was positively correlated with the IL-6 level and HAQ results. All of these results are also consistent with those previously published by other authors [36–38].

After their diagnosis, all patients were treated with methotrexate and >50 % of these patients received low doses of GC. This treatment yielded a significant improvement of arthritis and physical function in most patients and a decline of acute phase reactants and of resistin, adiponectin, TNF-α, and especially IL-6 levels. In our opinion, the improvement in physical function and the recreational physical activity possibly contributed to the maintenance of normal insulin sensitivity after 6 months of treatment. In addition, methotrexate is independently associated with a reduced propensity for metabolic syndrome, thus suggesting a drug-specific mechanism [39].

In contrast, GC use did not influence IR or adipokine levels in our patients after 6 months. This may be due to the complex relationship between GC and IR; in the short term perhaps, the potent anti-inflammatory effects of GCs overcome their adverse effects on body composition and glucose metabolism [40, 41]. We believe that these results reinforce the importance of an early diagnosis and treatment of RA, especially with methotrexate and a short course of low-dose GC, because these could prevent the long-term deleterious consequences of RA on both the joints and metabolism. Therefore, our results do not contradict, but instead complement those observed in patients with longstanding RA.

Thus far, no longitudinal study has investigated the effects of DMARDs on adipokine levels in early treatment-naive RA. Instead, there have only been a few studies in a small number of chronic RA patients [35, 42]. Our results are consistent with those observed after an effective suppression of inflammation during anti-TNF therapy [43, 44] but are inconsistent with those observed after several types of DMARD treatment [35, 42]. Nevertheless, these authors do not provide data on the patients’ response to DMARD treatment. Six months after treatment initiation, the concentrations of cytokines and adipokines significantly changed in our patients.

Resistin was the only adipokine that was elevated in RA patients, as has also been observed in other studies [36]. With regard to lipid levels, several studies have reported that patients with ERA are characterized by an atherogenic lipid profile that improves after treatment [45]. In the present study, after 6 months of treatment, an improved lipid profile was observed in accordance with earlier studies [45].

Although a larger sample size might have been helpful, the enrollment of ERA patients who were never exposed to DMARDs or GC was an already difficult task.

Moreover, although the use of HOMA instead of the hyperinsulinemic euglycemic clamp method may seem as a limitation, indirect methods such as HOMA and QUICKI have been validated for use. They are reliable indices and clamp substitutes for measuring IR in epidemiological studies, clinical trials, and clinical practice [46].

In conclusion, our data suggested that IR was not present in ERA patients at diagnosis either 6 months after treatment. However, the symptom duration and fat mass appeared to show a direct relationship. Similarly, early treatment of RA with methotrexate might prevent a further increase of IR.
